# Modulation of Fibrosis in Systemic Sclerosis by Nitric Oxide and Antioxidants

**DOI:** 10.1155/2012/521958

**Published:** 2011-10-31

**Authors:** Audrey Dooley, K. Richard Bruckdorfer, David J. Abraham

**Affiliations:** ^1^Centre for Rheumatology and Connective Tissue Disease, University College London Medical School, Royal Free Campus, London NW3 2PF, UK; ^2^Institute of Structural and Molecular Biology, University College London Medical School, Gower Street, London WC1E 6BT, UK

## Abstract

Systemic sclerosis (scleroderma: SSc) is a multisystem, connective tissue disease of unknown aetiology characterized by vascular dysfunction, autoimmunity, and enhanced fibroblast activity resulting in fibrosis of the skin, heart, and lungs, and ultimately internal organ failure, and death. One of the most important and early modulators of disease activity is thought to be oxidative stress. Evidence suggests that the free radical nitric oxide (NO), a key mediator of oxidative stress, can profoundly influence the early microvasculopathy, and possibly the ensuing fibrogenic response. Animal models and human studies have also identified dietary antioxidants, such as epigallocatechin-3-gallate (EGCG), to function as a protective system against oxidative stress and fibrosis. Hence, targeting EGCG may prove a possible candidate for therapeutic treatment aimed at reducing both oxidant stress and the fibrotic effects associated with SSc.

## 1. Introduction: Nitric Oxide and Systemic Sclerosis

The free radical nitric oxide (NO) is an important physiological signalling molecule, potent vasodilator, and mediator of oxidative stress. Nitric oxide is synthesised from L-arginine by NO synthase (NOS), and three main isoforms of NOS have been identified with a constitutive expression in neuronal (nNOS or NOS 1), endothelial (eNOS or NOS 3), and several other cell types including fibroblasts. Furthermore, an inducible expression (iNOS or NOS 2) in response to a variety of inflammatory stimuli is possible [1]. Although NO is a gas, it is a highly reactive, short-lived, molecule able to rapidly diffuse across cell membranes. Nitric oxide exerts its biological effects by the reaction of NO with a diverse range of targets such as haem groups, iron and zinc clusters, and cysteine residues. Since the discovery of NO as a key endothelial-derived vasodilator molecule in cardiovascular physiology and the award of the Nobel Prize in 1998 to Robert F. Furchgott, Ferid Murad, and Louis J. Ignarro, the field of NO research has rapidly expanded to encompass many biomedical areas. Subsequently, NO has been demonstrated to act as a signalling molecule in many other tissues and regulates physiological and cellular processes in a variety of pathologies such as hypertension, cancer, diabetes, and male impotence [1].

In the disease scleroderma (SSc: systemic sclerosis), the metabolism of NO appears to be profoundly disturbed. There is considerable evidence implicating overproduction of NO [2–5] and reactive oxygen species (ROS) such as superoxide anions (O_2_^.−^) and peroxynitrite (ONOO^−^) [3, 4, 6–8] in the pathogenesis of SSc, an often fatal rheumatic disease of unknown aetiology. Regulation of NO by endogenous levels of the NOS inhibitor asymmetric dimethylarginine (ADMA) has also recently been proposed [9]. Major features of SSc are enhanced fibroblast activity, collagen overproduction, autoimmunity, and vascular dysfunction [10–12]. There are several classified clinical subgroups, including limited (lSSc) and diffuse (dSSc) cutaneous SSc, which reflect the nature of the disease in their degree of skin sclerosis, immunological profile, and microvascular dysfunction [13, 14]. Endothelial activation and damage are also an early part of this process [14–17]. The nature of the factors that induce endothelial dysfunction is still unclear; however, there are several serological biomarkers that reflect the vasculopathy of the disease. These include the vasoconstrictor endothelin [18], cell adhesion molecules such as selectin [19], anti-endothelial antibodies [20], and the vasodilator nitric oxide [2, 3, 5]. While indeed an early modulator of disease activity is thought to be oxidative stress, the etiology of events and role of NO remain unknown. Recent reports suggest that the abnormal production of ROS is linked to fibroblast activation by the increased expression of stimulatory serum autoantibodies to the platelet-derived growth factor receptor in SSc [21].

## 2. Fibrosis and Oxidative Stress in Systemic Sclerosis

In SSc the excessive connective tissue fibrosis is the most characteristic pathological manifestation of the disease [10]. The fibrosis is especially prominent in the diffuse cutaneous form of SSc, where excessive connective tissue accumulation is due to overproduction of the extracellular matrix by fibroblasts and myofibroblasts, activated by soluble factors such as transforming growth factor beta (TGF-*β*) [22–26] and connective tissue growth factor [27]. Myofibroblasts are a differentiated and activated form of fibroblast which have been shown to persist in SSc fibroblast cultures and are responsible for increased collagen synthesis and deposition. The molecular mechanisms underlying the origin of the myofibroblast are complex; however, they play a crucial role in wound healing and the development of fibrosis [28, 29]. The fibrotic process is most prominent in the skin, lungs, heart, gastrointestinal tract, kidney, tendons and ligaments, and endocrine glands; widespread perivascular fibrosis also occurs. Fibrotic damage to these affected organs accounts for much of the morbidity and mortality associated with SSc. 

The scleroderma phenotype at the cellular level has also been shown to be characterized by oxidative stress, an imbalance between the elevated level of ROS and/or the impaired function of the antioxidant defence system [30]. ROS usually include O_2_^.−^, hydrogen peroxide (H_2_O_2_), hydroxyl radicals (^.^OH), and ONOO^−^. The sources of oxidative stress in SSc are complex and interactive and likely to be due to ischaemic-reperfusion injury, dysregulated metabolism of the free radical NO [2–5], and generation of ROS by fibroblasts [6] and activated leukocytes such as monocytes via the NADPH oxidase system [31]. ROS have been demonstrated to be cell transducers of fibroblast proliferation [6], collagen-gene expression, and myofibroblast phenotype conversion in SSc [6, 8, 21]. During the 1990s–early 2000s clear evidence for oxidative stress in SSc emerged by the enhanced oxidation of lipids and lipoproteins (oxLDL) [32, 33], increased isoprostane production [34–37], and also the presence of modified, nitrated proteins in the plasma and skin [4, 7]. In SSc what is less certain is the exact stage at which increases in free radicals and other reactive species occur, the potential counteractive role of the antioxidant defence system or how both elements are linked to the main events of the disease such as the vascular abnormalities, and the increased synthesis of extracellular matrix, leading to fibrosis. Early studies demonstrated that there is reduced antioxidant capacity in SSc; plasma ascorbic acid (vitamin C), *α*-tocopherol, *β*-carotene, and selenium were found to be lower in patients than in controls [30, 32, 38]. Additionally, the beneficial effects of the antioxidant probucol in patients with Raynaud's phenomenon, a vascular pathology which is associated with SSc, have been shown [39]. Subsequently, antioxidant therapy was proposed as a possible treatment in SSc [30, 38, 39] and also in other diseases [40, 41] with the focus being on the antioxidants counteracting the ROS-induced endothelial damage and vasculopathy that occur. More recently, natural antioxidants such as polyphenols from green tea (Camellia sinensis) extracts, a popular beverage consumed worldwide, have attracted attention due to their potent antioxidant effects, particularly that of one component, (-)-epigallocatechin-3-gallate (EGCG). In this paper, we will discuss findings about the mechanisms of NO-mediated modulation of fibrosis as well as evidence suggesting that the dietary antioxidant EGCG could be a therapeutic target for SSc.

## 3. Nitric Oxide as a Regulator of the Fibrogenic Response

The versatile free radical NO has been implicated in the pathogenesis of SSc. In most biological situations NO is largely oxidized to nitrate (NO_3_^−^) and nitrite (NO_2_^−^), with the measurement of total nitrate and nitrite NO_(*x*)_ production, as well as ADMA levels, seen as a reflection of endothelial dysfunction in many diseases [1, 9, 42]. Early studies involving NO_(*x*)_ production in SSc have shown conflicting results [2, 3, 5, 43, 44]; however, the discrepancy in these results could be explained by differences in the degree of inflammatory disorder, disease subset, and treatment of the patients. Furthermore, modifications in the dietary NO_(*x*)_ intake were not attempted in many of these studies. Later on other groups [2, 4, 5, 45, 46] demonstrated evidence implicating overproduction of NO, with increased plasma NO_3_^−^/NO_2_^−^ levels, together with elevated nitration of plasma proteins, a marker of ONOO^−^ production [4]. Nitrated proteins [47, 48] are also found in the skin in SSc associated with sites of inflammation [3, 4]. There is a growing literature showing strong tissue localization of nitrotyrosine staining or increased levels of free and protein-bound nitrotyrosine in inflammatory diseases. For example, strong staining of nitrotyrosine has been found in lung sections from patients with lung injury [49], while significant amounts of 3-nitrotyrosine have been reported from patients with rheumatoid arthritis [50] and chronic renal failure [51, 52]. 

**Figure 1:**
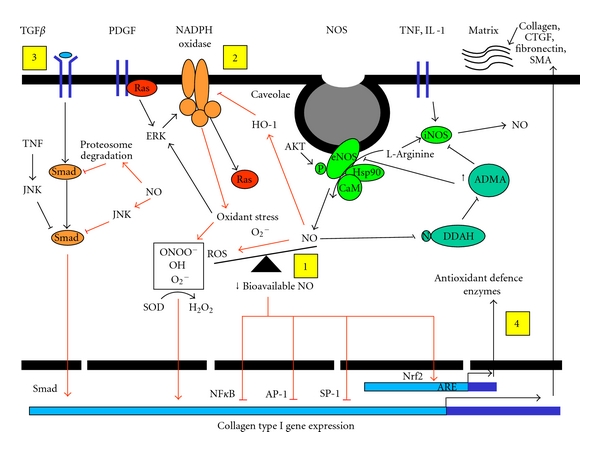
Schematic diagram depicting the possible pathways in which NO modulates collagen type I gene expression to affect fibrosis. In the first hypothesis (1), the rapid reaction between NO and O_2_^.−^ leads to decreased NO bioavailability. NO regulation by ADMA may also occur. NO normally can directly activate transcription factors such as NF*κ*B, SP-1, and AP-1 to inhibit collagen gene expression. The second possibility (2) is that NO normally by activating the protective stress enzyme HO-1 can negatively modulate the NADPH oxidase pathway. In fibrosis, activation of the NADPH oxidase pathway has been shown to increase collagen synthesis and myofibroblast differentiation. The third plausible pathway (3) is that there is signalling crosstalk following TGF-*β* binding to a receptor. Signal pathways potentially important here include the MAP kinase JNK. This would synergise with the Smad signalling pathway and decrease the activation of downstream TGF-*β*-dependent genes. Alternatively, NO could enhance the proteasomal degradation of SMAD. In the fourth pathway (4), NO indirectly exerts its effects by modulating oxidative stress through upregulation of antioxidant/redox defence genes such as Nrf2 leading to regulation of the extracellular matrix.

Interestingly, in SSc there is the paradoxical situation in which NO production by eNOS in endothelial cells is decreased possibly due to the rapid reaction of NO and O_2_^.−^ to generate the reactive intermediate ONOO^−^ [53, 54] or due to the presence in the circulation of natural inhibitors of NOS activity such as ADMA [9, 42] (Figure 1). Therefore, in SSc reduced eNOS expression and microcirculatory dysfunction are in part contributory to the associated Raynaud's Phenomenon that is well described in these patients [3, 14–16, 55]. In contrast at inflammatory sites the formation of NO, by iNOS, and O_2_^.−^ are increased by the presence of inflammatory cells such as macrophages or activated fibroblasts [56]. Immunohistological studies of scleroderma skin also show that, as the disease progresses to the later fibrotic stages, the production of eNOS is downregulated, while iNOS is upregulated [3]. Furthermore, studies suggest that under conditions when NO overproduction occurs, S-nitrosylation of the ADMA regulating enzyme dimethylarginine dimethylaminohydrolase (DDAH) diminishes DDAH activity, leading to an accumulation of ADMA. Subsequently, NOS inhibition as a type of regulatory feedback mechanism may result [57] (Figure 1). Indeed, there is further evidence to indicate increased circulatory levels of ADMA in the serum of diffuse SSc patients, suggesting an NOS regulatory mechanism later on in the disease [4]. 

NO has also been reported to act as an antifibrotic effector in animal models of experimental fibrosis [58, 59]. For example, in a murine model of pulmonary fibrosis, a loss of NO bioactivity, in eNOS knock-out mice, resulted in prolonged fibrosis [58]. Furthermore, another study also demonstrated that overexpressing eNOS, using transgenic mice, reduced fibrotic content after bleomycin-induced fibrosis [59]. In rats, the long-term inhibition of the inducible form of NOS (iNOS) has also been shown to favour the development of fibrosis [60]. Additional studies assessing NO metabolism in the tight-skin 1 (Tsk-1/^+^) mouse, which is predisposed to SSc and often used as an experimental animal model for fibrosis, reported that while type I collagen protein expression was elevated in Tsk-1/^+^ skin tissue, eNOS protein and gene expressions were reduced compared to wild-type controls [61]. Furthermore, there was decreased NOS activity in Tsk-1/^+^ skin tissue [61]. Correspondingly, the protective antioxidant enzyme haemoxygenase-1 (HO-1) and the associated transcription factor nuclear factor erythroid-2-related factor 2 (Nrf2) showed reduced protein and gene expression levels in Tsk-1/^+^ skin, while there was also less total antioxidant activity [61]. The findings suggested that there was also abnormal NO metabolism in the Tsk-1/^+^ mouse, particularly in the skin, while expression and activity of protective antioxidants were reduced. 

Several studies have gained insights into the intracellular mechanisms by which NO might further modulate a fibrotic phenotype. Nitric oxide can induce HO-1, which is also induced by a variety of cellular stress, including free-radical-mediated stresses and oxygen deprivation [62]. HO-1 in turn can also increase oxidative-stress-related transcription factors such as Nrf2. Because of high similarity between the Nrf2-binding sequence (NF-E2 motif) and the antioxidant response element (ARE) in regulatory regions of several phase 2 antioxidant enzymes, Nrf2 is a putative mediator of ARE responses. Consequently, other ARE-bearing protective phase 2 antioxidant/redox enzymes such as glutathione peroxidase, and classical antioxidant enzymes such as superoxide dismutase [SOD] and catalase as well as HO-1 induce Nrf2 gene expression (Figure 1). There is a large body of evidence suggesting that HO-1 is a cytoprotective enzyme, and its induction in the setting of increased cellular stresses helps maintain physiological homeostasis [63]. Thus, embryonic fibroblasts derived from HO-1^−/−^ knock-out mice are significantly less resistant to the cytotoxicity induced by H_2_O_2_ and paraquat than wild-type controls [64, 65]. Conversely, cells overexpressing HO-1 have been reported to be more resistant to oxidant-induced toxicity than controls [64]. Indeed, other groups which examined Nrf2^−/−^ knock-out mice reported increased bleomycin-induced pulmonary fibrosis [66] and expression of extracellular matrix genes such as collagens after hyperoxic exposure [67] when compared to wild-type controls. It has, thus, been suggested that an increased oxidative burden by suppression of antioxidant defence mechanisms in Nrf2^−/−^ mice secondarily triggers regulation of extracellular matrix genes for repair responses [67]. Interestingly, recently studies in SSc have also suggested Nrf2 as a target for antifibrotic therapy [68].

Other studies suggest that NO influences fibrosis through modulatory effects on the TGF-*β* pathway [69–71], initiation of fibroblast apoptosis and myofibroblast differentiation [60, 72, 73], and/or neutralization of profibrotic ROS [53, 54] (Figure 1). Additionally, NO can modulate collagen synthesis; however, at present, the mechanisms and signalling pathways of NO-mediated inhibition of collagen are not clear. NO inhibition of collagen in dermal SSc fibroblasts has been reported to be by cGMP-independent regulatory mechanisms and in part may be due to up-regulation of matrix metalloproteinase-1 (MMP-1, an essential collagenase involved in collagen degradation) protein and activity levels and/or inhibition of prolyl hydroxylase activity (an enzyme important in the posttranslational processing of collagen) [74]. Similarly, further evidence has also confirmed a role for NO in regulation of the extracellular matrix in other fibrotic diseases and cell types [70, 73, 75–80]. It was initially discovered in vascular smooth muscle cells [77, 78] and in mesangial cells [70, 80]. Recently, this downregulation has also been found in human dermal [69, 74], intestinal [81], and rat cardiac [76] fibroblast cells. From the view of the signalling pathway, it has been shown that NO downregulation of collagen synthesis in other cell types can occur in a cGMP-dependent [69, 76] or independent manner even though addition of NO could increase intracellular levels of cGMP [75, 82]. Furthermore other investigators using rat mesangial cells show that it is possible that the suppression of collagen synthesis by NO could involve an increase in MMP production and activity [80, 83]. Indeed, NO regulation of prolyl hydroxylase has been postulated in lapine articular chondrocytes [75]. Prolyl hydroxylase catalyses the formation of 4-hydroxyproline in collagens by hydroxylation of proline, and the reaction, in particular, requires Fe^2+^ and ascorbate and generates free radicals, all of which are sensitive to NO [84]. Under-hydroxylated procollagens do not form stable triple helices at body temperature and, thus, remain partially unfolded, where they are presumably more susceptible to degradation by intracellular collagenases. An additional number of intracellular targets for NO have been described [85], including NO regulation of NADPH oxidase [86], MAP kinases (e.g., JNK, ERK) [87], SMADs [71], and transcription factors (e.g., NF*κ*B, AP-1, SP-1) [88] which are key signalling pathways of fibroblast proliferation and collagen gene expression [6, 8, 25, 89, 90] (Figure 1). Taken together these studies strongly suggest a definitive link between NO expression and modulation of fibrosis.

## 4. Antioxidants, (-)-Epigallocatechin-3-Gallate, and Systemic Sclerosis 

In systemic sclerosis clear evidence for oxidative stress has been shown by increased levels of O_2_^.−^ [6], antibodies against oxLDL [91], enhanced lipid peroxidation [32, 33], increased F2-isoprostanes [35–37], and increased circulatory levels of nitrotyrosine [4, 7]. Additionally, ROS-induced endothelial damage occurs as well as the underlying vasculopathy associated with SSc known as Raynaud's Phenomenon. Raynaud's Phenomenon is characterised by transient attacks of cold-induced digital ischaemia associated with intense vasospasm in the fingers [55, 92, 93]. It occurs as a primary condition and as secondary to SSc. Currently, the underlying disorder of Raynaud's Phenomenon is thought to be related to the abnormal regulation of peripheral vascular tone at the level of the digital microcirculation [55, 92, 93]. Although nearly all patients with SSc exhibit Raynaud's Phenomenon, it is still uncertain whether their pathogenesis is identical to patients with Raynaud's Phenomenon alone. Furthermore, in SSc, it is not only the digital and peripheral vessels that exhibit vasospasm but also the vessels of the internal organs [13]. The pathophysiology underlying the cold-induced vasospasm characteristic of Raynaud's Phenomenon remains confused [55, 92]. Initially, it was suggested that *α*_2_-adrenoceptors accounted for the supersensitivity [94]. Further studies found that *α*_1_-adrenoceptors appears to predominate in the physiological control of cold-induced digital vasoconstriction whereas both *α*_1_- and *α*_2_-adrenoceptors play an equal role in Raynaud's subjects [15]. However, selective antagonism of these receptor subtypes in both normal and Raynaud's subjects did not abolish vasoconstriction suggesting that nonadrenergic mechanisms may also contribute to this response. Other studies in the dorsal hand vein [95], digital arteries [96], as well as isolated gluteal subcutaneous resistance arteries [97] of primary Raynaud's subjects show an impairment of endothelium-dependent relaxation. 

Although the vasospastic condition in Raynaud's Phenomenon may arise as a result of a functional disturbance at the level of the vessel wall, it is possible that circulating factors in the blood that alter the release of endothelial cell mediators, such as prostacyclin, NO, endothelin, or increase ROS and oxidative stress may contribute to the disease [93]. Early clinical trials, however, indicate limited success in treatment of patients with Raynaud's Phenomenon secondary to SSc with antioxidants such as *α*-tocopherol or vitamin C, which did not decrease urinary markers of oxidative stress such as F(2)-isoprostanes nor improved microvascular perfusion after cold exposure [98, 99]. More hopeful has been the use of the potent antioxidant N-acetylcysteine which has been shown to improve the vascular symptoms of Raynaud's Phenomenon in patients with SSc [100, 101]. The type of antioxidant used, phase of the disease, and duration of use may be key factors in successful treatment therapies involving antioxidants in both SSc and the associated Raynaud's Phenomenon. 

Natural antioxidants, such as polyphenols from green tea extracts, are now being considered and investigated in particular (-)-epigallocatechin-3-gallate (EGCG). Other less active polyphenol constituents of green tea are believed to be (-)-epigallocatechin (EGC), (-)-epicatechin (EC), and (-)-epicatechin-3-gallate (ECG). The prominent antioxidant effects of EGCG derive from the phenol rings that can act as electron traps to scavenge free radicals, inhibit the formation of ROS such as O_2_^.−^ and ONOO^−^, and reduce oxidative stress [102, 103]. EGCG has a higher potent antioxidant capability than *α*-tocopherol or vitamin C [104], and has been demonstrated to be an effective inhibitor of oxidative-stress-induced protein tyrosine nitration during isolation of platelets [48, 105].

Studies have also shown that EGCG may directly inhibit molecular targets and regulate multiple signal transduction pathways such as MAP kinases (PI3-kinase, ERK) and transcription factors (Nrf2, NF-*κ*B, AP-1) and/or induce antioxidant enzymes such as HO-1 [106, 107]. In addition to its antioxidant properties, it has been shown to possess antifibrotic, anticancer, and anti-inflammatory activities regulating both TGF-*β* and PDGF-induced *α*1(I) collagen, fibronectin, *α*-smooth muscle actin (*α*-SMA), and proliferation in activated human and rat hepatic stellate cells [108–110], rat pancreatic cells [111, 112], human keloid fibroblasts [113], and SSc dermal fibroblasts [114]. Additionally, EGCG can counteract TGF*β*-induced ROS in human dermal fibroblasts from healthy controls, SSc patients, and in a dermal fibroblast cell line, indicating its potential effectiveness as an antioxidant to reduce oxidant stress in the disease scleroderma [114]. Interestingly, other studies have shown data that EGCG can beneficially inhibit ROS through attenuating NADPH oxidase expression [115, 116]. Furthermore, topical administration of EGCG has successfully been shown to inhibit ultraviolet radiation-induced oxidative stress and tumorigenesis in human and animal skin models [117, 118]. Particularly useful will also be animal models of fibrosis where recently, in the case of bleomycin-induced pulmonary fibrosis and carbon tetrachloride-induced hepatic fibrosis, EGCG has been promisingly shown to exert anti-fibrotic effects [119–121]. 

In summary, it is clear that further studies are needed to delineate the key NO-mediated signal transduction and transcription pathways that facilitate type I collagen production and fibrosis in the disease scleroderma. Studies such as these will help define key targets and candidates for therapy. Furthermore the dietary antioxidant EGCG, with its long history of safe beverage consumption in green tea together with its demonstrated potent antioxidant capability, is a good candidate for therapeutic treatment targeting oxidative stress and fibrogenesis in patients with SSc. Further clinical studies, to confirm its efficacy, determine optimal dosage and duration of use, and treatment indicators are required.
